# The Effects of Clinical Decision Support Systems on Medication Safety: An Overview

**DOI:** 10.1371/journal.pone.0167683

**Published:** 2016-12-15

**Authors:** Pengli Jia, Longhao Zhang, Jingjing Chen, Pujing Zhao, Mingming Zhang

**Affiliations:** 1 Chinese Evidence-based Medicine Centre, West China Hospital, Sichuan University, Chengdu, PR, China; 2 Department of Otolaryngology-Head and Neck Surgery, The First Affiliated Hospital of Zhejiang Chinese Medical University, Hangzhou, PR, China; Cardiff University, UNITED KINGDOM

## Abstract

**Background:**

The clinical decision support system(CDSS) has potential to improving medication safety. However, the effects of the intervention were conflicting and uncertain. Meanwhile, the reporting and methodological quality of this field were unknown.

**Objective:**

The aim of this overview is to evaluate the effects of CDSS on medication safety and to examine the methodological and reporting quality.

**Methods:**

PubMed, Embase and Cochrane Library were searched to August 2015. Systematic reviews (SRs) investigating the effects of CDSS on medication safety were included. Outcomes were determined in advance and assessed separately for process of care and patient outcomes. The methodological quality was assessed by Assessment of Multiple Systematic Reviews (AMSTAR) and the reporting quality was examined by Preferred Reporting Items for Systematic Reviews and Meta-Analyses (PRISMA).

**Results:**

Twenty systematic reviews, consisting of 237 unique randomized controlled trials(RCTs) and 176 non-RCTs were included. Evidence that CDSS significantly impacted process of care was found in 108 out of 143 unique studies of the 16 SRs examining this effect (75%). Only 18 out of 90 unique studies of the 13 SRs reported significantly evidence that CDSS positively impacted patient outcomes (20%). Ratings for the overall scores of AMSTAR resulted in a mean score of 8.3 with a range of scores from 7.5 to 10.5. The reporting quality was varied. Some contents were particularly strong. However, some contents were poor.

**Conclusions:**

CDSS reduces medication error by obviously improving process of care and inconsistently improving patient outcomes. Larger samples and longer-term studies are required to ensure more reliable evidence base on the effects of CDSS on patient outcomes. The methodological and reporting quality were varied and some realms need to be improved.

## Introduction

Various interventions using information technology (IT) have been developed to improve medication safety, and IT-based interventions such as clinical decision support system(CDSS) plays an integral role in this field[1]. According to the definition of the US Food and Drug Administration, medication safety means risk management, medication errors, and surveillance for adverse drug reactions[2]. Medication errors are recognized as the single most preventable cause of patient harm, and their reduction is of increasing importance [1]. CDSS used to promote medication safety by facilitating evidence-informed medication use[3], reducing the incidence of harmful medication errors[4], and improving healthcare system efficiency[5]. A CDSS has been defined as a computerized system that uses case-based reasoning to assist clinicians in assessing disease status, in making a diagnosis, in selecting appropriate therapy or in making other clinical decisions[6].Characteristics of individual patients are matched to a computerized knowledge base, and software algorithms generate patient-specific information in the form of assessments or recommendations[7]. CDSS can improve medication safety and reduce medication-related expenditures because it encompasses a wide range of computerized tools directed at improving patient care, including computerized reminders and advice regarding drug selection, dosage, interactions, allergies, and the need for subsequent orders[8].

Medication errors and adverse drug events (ADEs) are common costly and clinical important problems[8]. More than half a million patients are injured or die each year in hospital from adverse drug events, which may cost up to USD 5.6 million annually per hospital in America[9]. Medication errors could occur at any stage of the medication management process, including prescription, transcription, preparation and administration[10]. A review identifying that prescribing errors occur in up to 11% of all prescriptions[11]. Two inpatient studies found that medication errors occurred at rates of more than 5% and nearly half of all medication errors occurred at the stage of drug ordering [8, 12]. Analysis of medication error suggests that prevention strategies targeting systems rather than individuals are most effective in reducing errors [13]. CDSS has been widely promoted as the most promising approaches that target the ordering stage of medications, where most medication errors and preventable ADEs occur [8].

Recently, several systematic reviews (SRs) have summarized the effects of CDSS on practitioner performance and patient outcome [7, 14–17] and the evidence has been synthesized into an overview[18].The overview demonstrated modest benefits on practitioner performance and patient outcomes [10]. Meanwhile, concerns have arisen regarding the impact of CDSS on medication safety and the evidence has been synthesized into several SRs, though findings from various SRs are conflicting. For example, a CDSS team conducted three SRs to evaluate the impact of CDSS on drug prescribing and management[4], medication dosing assistants[19] and therapeutic drug monitoring and dosing[20]; Two SRs summarized the effects of CDSS on ADEs[21] and medication safety[8]. Given the growing awareness of the importance of CDSS on medication safety and the numerous SRs on this topic, it is surprising that the overviews of the effects of CDSS in this field are lacking. As reported by Brown2014[22], overview brings SRs together into one coherent document and serve as a user-friendly “digest” by evaluating and synthetizing current evidence which can be used by clinicians and policy makers in making decisions about optimal treatment, so for the first time, we set out to conduct an overview to examine CDSS interventions on medication safety in health care settings. The purpose of our study is: to evaluate the effects of CDSS on medication safety; to examine the methodological and reporting quality and to highlight areas where more research is needed.

## Methods

### Inclusion criteria

#### Types of participants

The participants should be health care professionals such as: physicians, nurses, pharmacists, and other practitioners with responsibility for patient care. We excluded practitioners who are indirectly involved in patient care at ancillary clinical departments such as radiology, pathology departments.

#### Types of interventions

The CDSS combining clinical knowledge with patient characteristics and can provide either basic (e.g., drug-allergy checking) or advanced (e.g., drug dosing support for renal insufficiency) guidance to the participants.

#### Outcome measures

Within the SRs, at least one outcome relating to medication safety should be measured, such as: medication error, adverse drug events, prescribing errors, dosing errors and medication/drug related outcomes.

#### Selection of studies

Only SRs were considered for inclusion. To assess whether a study was a SR, we used the checklist of Cochrane Handbook for Systematic Reviews of Interventions [23].Two reviewers independently scanned titles and abstracts to exclude obviously irrelevant studies and potentially relevant studies were investigated as full text. Disagreements were resolved by discussion with a third reviewer.

### Search strategy

To identify relevant SRs, we searched the PubMed, Embase and Cochrane Library up to Aug. 2015 and elected English-only publications. Multiple keywords and Medical Subject Headings terms for CDSS were used. The search terms were customized for different databases (see S1 File: Search Strategy). No limitations were made on the outcomes. Additionally, we also handsearched reference lists and relevant reviews to identify SRs.

### Data extraction and management

Two reviewers independently extracted the data. We developed a data extraction sheet, pilot-tested it on 5 randomly-selected included studies, and refined it accordingly. The following data were extracted: general information, study design, study population, intervention and main study outcomes. Separate summaries were made for the outcomes. Within these summaries, the outcomes were determined in advance and assessed separately for process of care and patient outcomes (Table 1). A process of care outcome represents quality of care, such as practitioner performance and clinical intermediate outcome. A patient outcome is directly measured patient’s health and always be endpoint outcome, such as the number of symptomatic hypoglycaemic episodes, death or bleeding[20]. Results on process of care and patient outcomes were aggregated by grading them on the strength of evidence for improvement. The evidence strength is based on the included randomized controlled trials (RCTs) in the SRs[18]:

strong evidence: results based on RCTs and effect in 50% of more of the studies;

limited evidence: results based on RCTs and effect in 40–50% of the studies; results based on both RCTs and non-randomized studies and effect in 50% of the studies.

insufficient evidence: results based on non-randomized studies or effects in less than 40% of the studies.

**Table 1:** Definition of outcomes.

Term	Description
**Patient outcomes**	*1*.*endpoint outcome*: bleeding or thrombosis, hypoglycemic episodes, death, bleeding complications, adverse drug event, length of hospital stay, hospitalization
**Process of care outcomes**	*1*.*medication error(dose)*: drug dose, antibiotic dose, insulin dosing, change in the drug dose, excess dose error. 2.*medication error(prescription)*: changes in prescribing, prescription errors, appropriate prescription, medication administration errors, medication prescribing errors, medication errors of omission, alerting pharmacists to possible drug interaction, drug incompatibilities 3.*adherence*: physician compliance with alerts, physician compliance with reminders, adherence to guideline/recommendation, prescribing adherence, adherence to recommended vaccination, patient’ adherence to chronic medication 4.*intermediate outcome*: INR time in therapeutic range, serum concentration, time to achieve stabilization, physiological parameter change, blood glucose, physiological control, theophylline levels, time in target glucose range, time spent in target international normalized ratio, proportion of time in INR range, drug concentrations within desired range, digoxin serum aminoglycoside levels

### Quality assessment

We used two criteria sets for evaluating SRs because they have different foci. In the first stage, Assessment of Multiple Systematic Reviews (AMSTAR) was used to evaluate the methodological quality. AMSTAR is an 11-item measurement tool for the assessment of multiple systematic reviews that have good reliability and validity[24]. It was scored as “Yes”, “Partially yes”, “No”, “Can’t answer” or “Not applicable”. A criterion was defined as “Partially yes” if it was half met. For example, the fifth criterion, “A list of included and excluded studies should be provided”, was scored as “Partially yes” if either the included or the excluded studies were listed[25]. Each item is given a score of 1 if the specific criterion is met, a score of 0.5 if the criterion is partially met, or a score of 0 if the criterion is not met, is unclear, or is not applicable[24]. We summarized the final scores at three levels based on the criterion used by Beverley and Jeremy: 9 to 11 is high quality; 5 to 8 is medium quality and 0 to 4 is low quality[26].

In the second stage, the Preferred Reporting Items for Systematic reviews and Meta-Analyses (PRISMA) was used to evaluate the reporting quality. The checklist consists of 27 items and focuses on ways in which authors can ensure the transparent and complete reporting of systematic reviews and meta-analyses[27]. To indicate the degree of compliance, each checklist item was assigned one of four responses: ‘Yes’ for total compliance; ‘partial’ for partial compliance; ‘No’ for noncompliance and ‘NA’ for not applicable[28].

### Data analysis

We planned to conduct a meta-analysis if the treatment outcomes considered were comparable. Continuous measures were reported as mean differences and standard deviations or as standardized mean differences. Dichotomous outcomes were reported as odds ratio or rate ratio. Where studies provided sufficient data for meta-analysis, the Cochrane Collaboration’s RevMan software was used to perform a meta-analysis [23]. In studies in which outcome data were not suitable for meta-analysis, the data were described narratively.

## Results

### Search results

**Fig 1:**
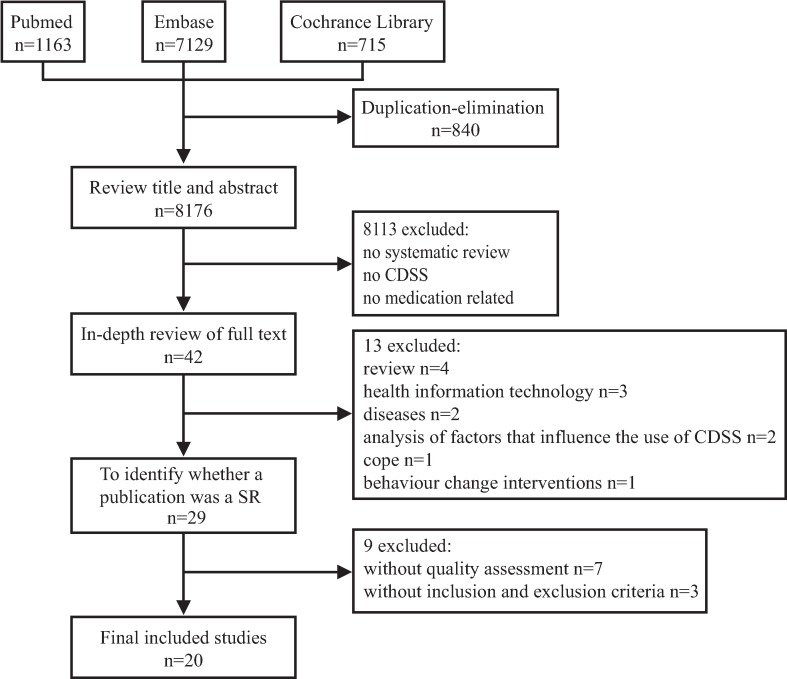
Flow Diagram for searching and selection processes.

The initial search provided a pool of 9,002 SRs published in English. After eliminating based on duplicates, title and abstract, 42 SRs remained for full texts sifting and 29 finally proved to fulfil the inclusion criteria. Then two reviewers independently assessed the 29 SRs using the checklist of handbook to determine whether a publication was a SR (Table 2, Table 3). Finally, 20 SRs were included (Fig 1).

**Table 2:** The checklist of Cochrane Handbook for Systematic Reviews of Interventions to identify SR.

ID	Items	Description
1	objective	to summarize evidence from studies of the effects of interventions
2	study selection	original studies, such as RCT, cross-over study
3	study plan	protocol
4	selection criteria	describe inclusion and exclusion criteria
5	search	comprehensive search processes for relevant studies
6	quality assessment	evaluate the quality of the studies in the systematic reviews.
7	analysis	meta-analysis or descriptive analysis
8	outcome	objectively describe the characteristics, quality assessment outcomes, the effect size and the publication bias of the included studies.
9	conclusion	comprehensively consider the quality, effect size and address the implications for future research.
10	reporting	reporting results according to the PRISMA guidelines

**Table 3:** Systematic review appraisal based on Cochrane Handbook for Systematic Reviews of Interventions.

Study ID	checklist of Cochrane Handbook for Systematic Reviews of Interventions									
	1	2	3	4	5	6	7	8	9	10
**Inclusion**										
1.Bayoumi2014^[^^31^^]^	Y	Y	Y	Y	Y	Y	Y	Y	Y	Unclear
2.Keers2014^[^^39^^]^	Y	Y	Y	Y	Y	Y	Y	Y	Y	Unclear
3.Gillaizeau2013^[^^30^^]^	Y	Y	Y	Y	Y	Y	Y	Y	Y	Unclear
4.Alldred2013^[^^38^^]^	Y	Y	Y	Y	Y	Y	Y	Y	Y	Unclear
5.Vervloet2012^[^^40^^]^	Y	Y	N	Y	Y	Y	Y	Y	Y	Unclear
6.Manias2012^[^^10^^]^	Y	Y	N	Y	Y	Y	Y	Y	Y	Unclear
7.Tawadrous2011^[^^32^^]^	Y	Y	Y	Y	Y	Y	Y	Y	Y	Yes
8.Sahota2011^[^^19^^]^	Y	Y	Y	Y	Y	Y	Y	Y	Y	Unclear
9.Nieuwlaat2011^[^^20^^]^	Y	Y	Y	Y	Y	Y	Y	Y	Y	Unclear
10.Hemens2011^[^^4^^]^	Y	Y	Y	Y	Y	Y	Y	Y	Y	Unclear
11.Loganathan2011^[^^37^^]^	Y	Y	N	Y	Y	Y	Y	Y	Y	Unclear
12.Robertson2010^[^^17^^]^	Y	Y	N	Y	Y	Y	Y	Y	Y	Unclear
13.Schedlbauer2009^[^^29^^]^	Y	Y	N	Y	Y	Y	Y	Y	Y	Unclear
14.Shojania2009^[^^35^^]^	Y	Y	Y	Y	Y	Y	Y	Y	Y	Unclear
15.Pearson2009^[^^34^^]^	Y	Y	N	Y	Y	Y	Y	Y	Y	Unclear
16.Amit2005^[^^16^^]^	Y	Y	N	Y	Y	Y	Y	Y	Y	Unclear
17.Bennett2003^[^^33^^]^	Y	Y	N	Y	Y	Y	Y	Y	Y	Unclear
18.Walton1999^[^^36^^]^	Y	Y	N	Y	Y	Y	Y	Y	Y	Unclear
19.Dereck1998^[^^15^^]^	Y	Y	N	Y	Y	Y	Y	Y	Y	Unclear
20.Johnston 1994^[^^14^^]^	Y	Y	N	Y	Y	Y	Y	Y	Y	Unclear
**Exclusion**										
21.Tran2014	Y	Y	N	Y	Y	N	Y	Y	Y	Unclear
22.Georgiou2013	Y	Y	Y	Y	Y	N	Y	Y	Y	Unclear
23.Yourman2008	Y	Y	N	Y	Y	N	Y	Y	Y	Unclear
24.Wolfstadt2008	Y	Y	N	N	Y	Y	Y	Y	Y	Unclear
25.Shebl2007	Y	Y	N	Y	Y	N	Y	Y	Y	Unclear
26.Conroy2007	Y	Y	N	Y	Y	N	Y	Y	Y	Unclear
27.Kaushal2003	Y	Y	N	N	Y	N	Y	Y	Y	Unclear
28.Fitzmaurice1998	Y	Y	N	N	Y	Y	Y	Y	Y	Unclear
29.Chatellier1998	Y	Y	N	Y	Y	N	Y	Y	Y	Unclear

Note: A publication was determined as a SR if meets the items (1, 2,4,5,6,7,8,9).

### General study characteristics

The 20 SRs were published between 1994 and 2014 and conducted in diverse and mixed study settings, such as: inpatient settings[16 17 29–31], outpatient settings[4 29 30 32], ambulatory settings[17 31 33 34], hospital setting[14 29 32] and primary medical setting[29 33], involving over 256,980 health care practitioners and 1,683,675 patients. All the studies were from developed countries, of which 9 studies were undertaken in Canada[4 14–16 19 20 31 32 35], followed by the United Kingdom[29 36–39], Australia[10 17 33 34],Netherlands[40]and France[30]. In total these SRs included 629 references with 401 RCTs. All studies evaluated multiple medications: anticoagulant[16 17 30 31 34 36], theophylline or aminophylline[15 16 20 30 31 36],insulin[20 30 31],aminoglycoside[19 20 36]. Fourteen studies reported funding sources and all were supported by public funding[4 10 14 16 17 19 20 32 34–39], while six studies did not report [15 29–31 33 40](Table 4).

**Table 4:** Levels of evidence for clinical decision-support systems (CDSS) impacting process of care and patient outcomes.

1.Study ID 2.Country	1.Population 2.Number	Setting	1.Intervention 2.Based Study design/NO.	targeted disease or medication	Outcome
**1.Bayoumi 2014**^**[**^^**31**^^**]**^ **2. Canada**	1.physicians, nurses, nurse practitioners, pharmacists, physician assistants, unspecified clinicians 2.79.273	ambulatory settings, inpatient settings, nursing homes, emergency department	1.computerized drug lab reminder systems 2.RCT/36	anticoagulation, antimicrobial, digoxin, insulin, theophylline, multiple drug-lab combinations	• **Process of care outcome** 1.medication error (prescription): pooling of results, improvement in 3 of 6 studies---SE 2.adherence: pooling of results, improvement in 4 of 8 studies---SE 3.intermediate outcome: improvement in 8 of 11 studies---SE • **Patient outcome** 1.endpoint outcome: improvement in 1 of 11 studies---IE
**1.Gillaizeau 2013**^**[**^^**30**^^**]**^ **2. France**	1.doctors, pharmacists, nurse behavior 2.41857	inpatient settings, outpatient settings, community mix	1.computerized advice on drug dosage 2.RCT/44, cluster RCT/2	anticoagulants, insulin, theophylline, anti-rejection drugs, infusions of anesthetics agents, amitriptyline study, gonadotropins	• **Process of care outcome** 1.intermediate outcome: improvement in 20 of 28 studies---SE • **Patient outcome** 1.endpoint outcome: improvement in 4 of 27 studies---IE
**1.Vervloet2012**^**[**^^**40**^^**]**^ **2. Netherlands**	1.adult patients, adult and adolescent patients, women on oral contraceptives 2.1536	any healthcare setting	1.electronic reminder 2.RCT parallel**/**10, RCT crossover/3	HIV, asthma, hypertension, glaucoma, oral contraceptives	• **Process of care outcome** 1.patients’ adherence to chronic medication: improvement in 8 of 13 studies---SE
**1.Sahota2011**^**[**^^**19**^^**]**^ **2. Canada**	1.physicians, trainees, advanced practice nurses pharmacists, other health professionals 2.3417 1.patients 2.202,491	121 different clinics at 106 sites	1.CDSS for medication dosing assistants 2.RCT/36	warfarin, aminoglycoside, oral anticoagulants, aminoglycoside, theophylline, others	• **Process of care outcome** 1.medication error (dose): improvement in 17 of 23 studies---SE 2.intermediate outcome: improvement in 6 of 8 studies---SE • **Patient outcome** 1.endpoint outcome: improvement in 2 of 3 studies---SE
**1.Nieuwlaat2011**^**[**^^**20**^^**]**^ **2. Canada**	1.physicians, other health professionals 2.1072 1.patients 2.24,627	the majority being performed at a single center	1.CCDSS for patient care 2.RCT/33	vitamin K antagonist, theophylline, aminophylline, insulin/glycemic regulation, aminoglycoside, digoxin, lidocaine	• **Process of care outcome** 1.medication error (dose): improvement in 1 of 1 studies---SE 2.adherence: improvement in 2 of 4 studies---SE 3.intermediate outcome: improvement in 11 of 22 studies---SE • **Patient outcome** 1.endpoint outcome: improvement in 3 of 20 studies---IE
**1.Hemens2011**^**[**^^**4**^^**]**^ **2.Canada**	1.fully-trained physicians, post-graduate medical trainees, nurses, physician assistants, pharmacists 2.8932 1.patients 2.246,686	outpatient settings, academic settings, outside academic centers	1.a group of providers or patientsusing a CCDSS 2.RCT/65	cardiovascular disease, diabetes mellitus, respiratory disease, dyslipidaemia, infectious diseases	• **Process of care outcome** 1.medication error(prescription): improvement in 20 of 28 studies---SE 2.medication error(dose): improvement in 3 of 6 studies---SE 3.adherence: improvement in 9 of 17 studies---SE 4.intermediate outcome: improvement in 4 of 8 studies---SE • **Patient outcome** 1.endpoint outcome: improvement in 1 of 11 studies---IE
**1.Tawadrous2011**^**[**^^**32**^^**]**^ **2. Canada**	1.health care providers 2.unknown	hospital setting, outpatient setting, across several facilities	1.computerized or manual CDSS 2.RCT/5, alternating time-series/2, cohort with historical controls/16, cohort with no controls/8, cohort with concurrent controls/3	NDD, decreased kidney function, end-stage kidney disease, kidney transplant recipients	• **Process of care outcome** 1.medication error(dose): improvement in 25 of 27 studies (93%)---LE 2.intermediate outcome: improvement in 1 of 5 studies (20%)---IE • **Patient outcome** 1.endpoint outcome: 1 study, no evidence of improvement---IE
**1.Robertson2010**^**[**^^**17**^^**]**^ **2. Australia**	1.pharmacists, physicians, nurses, nurse practitioners 2.105399	ambulatory care, hospital inpatients	1.computerized or paper-based CDSS 2.RCT/16, non-randomized studies with concurrent or historical control groups/4, interrupted time-series design/1	cardiovascular disease, anticoagulant therapy, antibiotic therapy, respiratory conditions, diabetes elderly, renal impairment	• **Process of care outcome** 1.medication error(prescription): improvement in 9 of 16 studies (56%)---SE 2.adherence: improvement in 1 of 3 studies (33%)---IE 3.pharmacist activity: improvement in 2 of 2 studies (100%)---SE 4.intermediate outcome: improvement in 1 of 3 studies (33%)---IE • **Patient outcome** 1.endpoint outcome: improvement in 3 of 6 studies(50%)---LE
**1.Shojania2009**^**[**^^**35**^^**]**^ **2. Canada**	majority of participants (> 50%) consisted of physicians or physician trainees	any healthcare setting	1.a reminder delivered via a computer system 2.RCT**/**32	antibiotics, asthmas, aspirin, diabetes, hypertension, erythropoietin, hemoglobin	• **Process of care outcome** 1.adherence: improvement in 27 of 32 studies(84%)---SE
**1.Amit2005**^**[**^^**16**^^**]**^ **2. Canada**	1.practitioners or practices 2.3826 1.patients 2.92895	academic centers, inpatient-based	1.CDSS for patient care 2. randomized trials/88, nonrandomized/12	anticoagulant, theophylline, aminophylline, asthma, hypertension	• **Process of care outcome** 1.medication error (dosing and prescribing): improvement in 7 of 11 studies (64%)---LE 2.intermediate outcome: improvement in 8 of 13 studies (62%)---LE • **Patient outcome** 1.endpoint outcome: improvement in 12 of 16 studies(75%)---LE
**1.Bennett2003**^**[**^^**33**^^**]**^ **2. Australia**	1.physician, nurse 2.1823 1.patients 2.15732	general medicine, primary medical, ambulatory care	1.computer assist system in identifying patients and generating reminders or feedback 2.RCT**/**26	aspirin, antacid, digitalis, metronidazole	• **Process of care outcome** 1.medication management: improvement in 19 of 22 studies (86%)---SE 2.patient adherence to medication improvement in 3 of 22 studies(14%)---IE
**1.Walton1999**^**[**^^**36**^^**]**^ **2. UK**	1.quantitative analysis was based on results derived from only 671 patients	any healthcare setting	1.computer aided decisions computer directly administered the drug to patients 2.RCT**/**16, nonrandomized controlled clinical trial/1	anesthesia, anticoagulation, aminoglycosides, theophylline	• **Process of care outcome** 1.medication error(dose): pooling of results, improvement in 7 of 11 studies (64%)---LE 2.medication error(prescription): improvement in 9 of 16 studies (56%)---SE 3.intermediate outcome: improvement in 4 of 15 studies (26%)---IE 4.outcome of medical care: improvement in 5 of 6 studies (83%)---SE • **Patient outcome** 1.endpoint outcome: improvement in 3 of 5 studies(60%)---SE
**1.Dereck1998**^**[**^^**15**^^**]**^ **2. Canada**	1.patients 2.91456	clinical setting	1.CDSS evaluated in clinical setting 2.trials randomized (majority), quasi-random/ 9	aminophylline, warfarin, theophylline, intravenous medications, hypertension	• **Process of care outcome** 1.medication error(dose): improvement in 6 of 8 studies (75%)---SE • **Patient outcome** 2.endpoint outcome: improvement in 1 of 4 studies (25%)---IE
**1.Johnston1994**^**[**^^**14**^^**]**^ **2. Canada**	1.nurse/ physician team, physicians alone, nurses alone	teaching hospital and affiliated clinic	1.a computer based CDSS evaluated in a clinical setting 2.controlled trials/ 28	toxic drugs, blood pressure, hypertension, vaccination	• **Process of care outcome** 1.medication error(dose): improvement in 0 of 4 studies (0%)---IE
**1.Pearson2009**^**[**^^**34**^^**]**^ **2. Australia**	1.physicians (35 studies) 1.physicians with medical students and/or other health care, professionals (21 studies) 2.unkonw	ambulatory care, institutional care	1.computerised CDSS to routine care and/ or paper-based decision support 2.RCT**/**50, quasi-experiments/6	cardiovascular disease, antibiotic therapy, vaccinations, respiratory conditions, anticoagulant therapy, elderly, osteoporosis	• **Process of care outcome** 1.medication error(prescribing): improvement in 19 of 36 studies (52%)---LE 2.medication error(dose): improvement in 8 of 15 studies (53%)---LE
**1.Schedlbauer2009**^**[**^^**29**^^**]**^ **2. UK**	1.hospital doctors, nurses and nurse practitioners 2.unkonw	primary care, outpatient, hospital/inpatient	1.computerized drug alerts and prompts to clinicians’ prescribing behavior 2.interrupted time series analyses/ 4, time series analyses/ 6, before-after design/ 6	sedatives, lipid lowering drugs, asthma, antibiotic	• **Process of care outcome** 1.medication error(prescribing): improvement in 4 of 4 studies (100%)---LE 2.prescribing behavior: improvement in 25 of 27 studies (93%)---LE • **Patient outcome** 1.patients’ medical conditions: improvement in 5 of 8 studies(63%)---LE
**1.Keers2014**^**[**^^**39**^^**]**^ **2. UK**	1.nurse, anesthetist 2.unkonw	medical settings, surgical, intensive care settings, step-down units, operating theatre geriatric assessment and rehabilitation	1.any intervention(s) on the rate of MAEs 2.RCT**/**6, non-randomized controlled trial/7	medication administration errors (timing errors, wrong dose)	• **Process of care outcome** 1.medication error(medication administration errors): pooling of results, improvement in 3 of 3 studies---LE
**1.Alldred2013**^**[**^^**38**^^**]**^ **2. UK**	1.residents 2.7653	older people (aged 65 years or older) living in institutionalized care facilities	1.interventions concerned with optimizing the whole medication regime 2.RCT/8	adverse drug events, preventable adverse drug events	• **Patient outcome** 1.endpoint outcome: improvement in 0 of 1 study---IE
**1.Manias2012**^**[**^^**10**^^**]**^ **2. Australia**	1.patients 2.sample sizes ranged from 25 to 8901 patients	ICU for adult patients	1.intervention in intensive care for adult patients with the aim of reducing medication errors were 2.pre-post interventional studies/22, prospective randomized trials/2	medication errors	• **Process of care outcome** 1.medication error(dose): improvement in 3 of 3 studies (100%)---LE 2.medication error(prescribing): pooling of results, improvement in 3 of 3 studies (100%)---LE 3.intermediate outcome: pooling of results, improvement in 1 of 1 study (100%)---LE 4.number of medication incidents: improvement in 1 of 1 study(100%)---LE
**1.Loganathan2011**^**[**^^**37**^^**]**^ **2. UK**	1.residents’ mean age≥65 2.3728	nursing homes, residential homes, long-term care facilities, mixed home	1.intervention on prescribing, aimed at improving appropriate prescribing 2.cluster RCT/11 (2 about CDSS), RCT/2, controlled before-and-after study/2, before-and-after study with additional, post-intervention concurrent control/1	antidepressant, antihypertensive, hypnotics, warfarin, aspirin, antipsychotic	• **Process of care outcome** 1.practitioner performance 2.medication error(dose): pooling of results, improvement in 1 of 1 study (100%)---SE • **Patient outcome** 1.endpoint outcome: no effect in 1 study---IE
					

Note: CDSS: clinical decision support system, RCT: randomized controlled trial, NDD, non–dialysis dependent, IE, insufficient evidence; LE, limited evidence; SE, strong evidence

### Quality assessment

#### Methodological quality

Ratings for the overall scores of AMSTAR resulted in a mean score of 8.3 with a range of scores from 7.5 to 10.5. Based on the criterion used by Beverley and Jeremy[26]: four studies is high quality [20 30 36 38]; Sixteen is medium quality [4 10 14–17 19 29 31–35 37 39 40] and 0 to 4 no study is low quality (Table 4). Meanwhile, the 20 SRs varied in the tools used to assess the methodological quality of their including studies: 10-point scale was used by six studies[4 16 19 20 32 34]; Two SRs conducted the processes based on the criteria developed by the Cochrane Effective Practice and Organization of Care (EPOC) Group [30 39]; The Cochrane Collaboration’s tool for assessing risk of bias was used by Bennett2003[33] and Alldred2013[38] and one study used the Downs and Black tool[37] (Table 5).

**Table 5:** The results of methodological quality based on AMSTAR.

Study ID	AMSTAR criteria											AMSTARscore
	1	2	3	4	5	6	7	8	9	10	11	
1.Bayoumi2014^[^^31^^]^	Y	P	P	P	P	Y	Y	Y	Y	N	Y	8
2.Keers2014^[^^39^^]^	Y	Y	Y	Y	P	Y	Y	Y	NA	NA	Y	8.5
3.Alldred2013^[^^37^^]^	Y	Y	Y	Y	Y	Y	Y	Y	NA	NA	Y	9
4.Gillaizeau2013^[^^30^^]^	Y	Y	Y	P	Y	Y	Y	Y	Y	Y	Y	10.5
5.Manias2012^[^^10^^]^	Y	Y	P	P	P	Y	Y	Y	Y	NA	Y	8.5
6.Vervloet2012^[^^40^^]^	Y	Y	Y	P	P	Y	Y	Y	NA	NA	Y	8
7.Loganathan2011^[^^37^^]^	Y	Y	Y	P	P	Y	Y	Y	NA	NA	Y	8
8.Sahota2011^[^^19^^]^	Y	Y	Y	P	P	Y	Y	Y	NA	NA	Y	8
9.Nieuwlaat2011^[^^20^^]^	Y	Y	Y	Y	P	Y	Y	Y	Y	NA	Y	9.5
10.Tawadrous2011^[^^32^^]^	Y	Y	Y	P	P	Y	Y	Y	NA	NA	Y	7.5
11.Hemens2011^[^^4^^]^	Y	Y	Y	P	P	Y	Y	Y	NA	NA	Y	8
12.Robertson2010^[^^17^^]^	Y	Y	Y	P	P	Y	Y	Y	NA	NA	Y	8
13.Shojania2009^[^^35^^]^	Y	Y	Y	P	Y	Y	Y	Y	NA	NA	Y	8.5
14.Schedlbauer2009^[^^29^^]^	Y	Y	P	P	P	Y	Y	Y	NA	NA	Y	7.5
15.Pearson2009^[^^34^^]^	Y	Y	Y	P	P	Y	Y	P	NA	NA	Y	7.5
16.Amit2005^[^^16^^]^	Y	Y	Y	Y	P	Y	Y	Y	NA	NA	Y	8.5
17.Bennett2003^[^^33^^]^	Y	Y	P	P	P	Y	Y	Y	NA	NA	Y	7.5
18.Walton1999^[^^36^^]^	Y	Y	P	P	Y	Y	Y	Y	Y	NA	Y	9
19.Dereck1998^[^^15^^]^	Y	Y	Y	Y	P	Y	Y	Y	NA	NA	Y	8.5
20.Johnston 1994^[^^14^^]^	Y	Y	P	P	P	Y	Y	Y	NA	N	Y	7.5

Note:Y:Yes; P:Partially yes; N: No; CA: Can’t answer; NA: Not applicable.

#### Reporting quality

Overall, none of the included 20 SRs fulfilled all 27 items of PRISMA. The “rationale for the review, study selection, definition of data items, synthesis of results, presentation of study characteristics, summary of evidence and conclusions” were well described across all SRs; The item “Identify the report as a systematic review, meta-analysis, or both” were adequately reported in 75% of the SRs. Search strategy for at least one major database was reported by 15 SRs; “Risk of bias of individual studies” were evaluated completely in 17 SRs. Total compliance with PRISMA items was less than 30.0% in structured summary (item 2, 5%), objectives (item 4, 15%), data collection process (item 10, 5%), summary measures (item 13, 25%), results of individual studies (item 20, 25%); No study described additional analyses in the methods, such as meta-regression or sub-group analyses (Table 6).

**Table 6:** The results of reporting quality assessment.

PRISMA items		Yes	Partial	No	NA
		n (%)	n (%)	n (%)	n (%)
**Title**	**1. Title**	15(75.0)	2(10.0)	3(15.0)	0(0)
**Abstract**	**2.Structure**	1(5.0)	19(95.0)	0(0)	0(0)
**Introduction**	**3. Rationale**	20(100.0)	0(0)	0(0)	0(0)
	**4. Objectives**	3(15.0)	17(85.0)	0(0)	0(0)
**Methods**	**5.Protocol and registration**	8(40.0)	0(0)	12(60.0)	0(0)
	**6. Eligibility criteria**	19(95.0)	1(5.0)	0(0)	0(0)
	**7. Information sources**	13(65.0)	7(35.0)	0(0)	0(0)
	**8. Search**	15(75.0)	5(25.0)	0(0)	0(0)
	**9. Study selection**	20(100.0)	0(0)	0(0)	0(0)
	**10. Data collection process**	1(5.0)	19(95.0)	0(0)	0(0)
	**11. Data items**	20(100.0)	0(0)	0(0)	0(0)
	**12. Risk of bias in individual studies**	17(85.0)	3(15.0)	0(0)	0(0)
	**13. Summary measures**	5(25.0)	0(0)	0(0)	15(75.0)
	**14. Synthesis of results**	20(100.0)	0(0)	0(0)	0(0)
	**15. Risk of bias across studies**	0(0)	1(5.0)	4(20.0)	15(75.0)
	**16. Additional analyses**	0(0)	0(0)	6(30.0)	14(70.0)
**Results**	**17. Study selection**	15(75.0)	5(25.0)	0(0)	0(0)
	**18. Study characteristics**	20(100.0)	0(0)	0(0)	0(0)
	**19. Risk of bias within studies**	18(90.0)	2(10.0)	0(0)	0(0)
	**20. Results of individual studies**	5(25.0)	15(75.0)	0(0)	0(0)
	**21. Synthesis of results**	19(95.0)	1(5.0)	0(0)	0(0)
	**22. Risk of bias across studies**	0(0)	0(0)	20(100.0)	
	**23. Additional analysis**	0(0)	0(0)	5(25.0)	15(75.0)
**Discussion**	**24. Summary of evidence**	20(100.0)	0(0)	0(0)	0(0)
	**25. Limitations**	17(85.0)	0(0)	3(15.0)	0(0)
	**26. Conclusions**	20(100.0)	0(0)	0(0)	0(0)
**Funding**	**27. Funding**	17(85.0)	0(0)	3(15.0)	0(0)

**Note:** NA: Not applicable.

### Synthesis of evidence

We planned to conduct a meta-analysis if the treatment outcomes considered were comparable. However, the variability in methods and the ways outcomes measured and presented made the generation of pooled estimates impossible. We presented the results in narrative and tabular form. The 20 SRs included 629 references, 413 of which represented unique studies, with 237 RCTs. Of these unique 237 RCTs, 178 RCTs studied process of care or patient outcomes. Nineteen of the 20 SRs examined the influence of CDSS on process of care: medication error (prescription)[4 10 17 29 31 34 36], medication error (dose)[4 10 15 16 19 20 32 34 36 37], adherence[4 17 20 31 33 35 40], medication or drug related intermediate outcome[4 10 16 19 20 30–32 36]. Evidence that CDSS significantly impacted process of care was found in 108 out of 143 unique studies of the 19 SRs that examined this effect (75%).Twelve of these 19 SRs found strong evidence that CDSS improved process of care: medication error (prescription)[4 17 31 36], medication error (dose)[4 15 19 20 37], adherence[4 20 31 35 40], medication or drug related intermediate outcome[4 19 20 30 31]; Seven of these 19 SRs found limited evidence that CDSS improved process of care: medication error (prescription)[10 29 34], medication error (dose^)^[10 16 32 34 36], medication or drug related intermediate outcome[10 16]; Six of these 19 SRs found insufficient evidence that CDSS improved process of care: medication error (dose)[14] adherence[17 33], medication or drug related intermediate outcome[32 36] (Table 4).

Thirteen out of the 20 SRs studied the impact of CDSS on patient outcomes. Evidence that CDSS significantly impacted patient outcomes was found in 18 out of 90 unique studies of the 13 SRs that examined this effect (20%).Only two of the 13 SRs found strong evidence that CDSS impacted patient outcomes: computer support for determining drug dose [36]and computerized medication dosing assistants [19] Three found limited evidence [16 17 29] and the remaining 8 SRs found insufficient evidence: computerized drug-lab alerts[31], computerized drug dose[15 20 30], computerized clinical decision support systems for drug prescribing[4 32 37 38] (Table 4).

## Discussion

Currently, an overview focuses on the effects of CDSS on practitioner performance and patient outcome has been published[18] and AMSTAR was used to evaluated the quality of the included SRs. To the best of our knowledge, this is the first overview focus on examining the effects of CDSS on medication error which is more important and direct on patient safety, specifically the drug-related injuries[21]. It is, furthermore, the first overview assessed the quality of SRs on the effectiveness of CDSS interventions using AMSTAR and PRISMA. AMSTAR focuses on methodological quality, while PRISMA incorporates items deemed important for the transparent reporting of a SR. Both the methodological and reporting quality are important for judging the overall strength of evidence on given research questions and are considered in assessment of the quality of a research design, implementation and reporting rather than the intervention’s true effect in the process of research[41]. The study by Spyridon demonstrated that the evaluation of the reporting quality of published SRs is very useful, as it is directly related to the study’s methodology and conclusions[25].

Our recent initial search yielded 9,002 SRs, of which only 20 SRs met inclusion criteria. We planned to examine the effects of CDSS on medication safety, four studies directly targeting medication error[10 39]or adverse drug events[37 38]. The majority of our studies (16 studies) focused on the medication/drug related outcome (i.e. INR time in therapeutic range, serum concentration, time to achieve stabilization, physiological parameter change, drug dose/prescribing). This situation was true in our study, but changes in medication/drug related outcomes are also important. A study by Lainer2013 showed that the CDSS interventions consistently improve medication/drug related outcome that may provide indirect evidence for the improvement of medication safety[1]. So the influence of CDSS on medication safety may be direct, or indirect. Although a surrogate measure, changes in accordance with best practice guidelines and underpinned by evidence from high-quality would be expected to deliver improved medication safety, even if the evidence was not captured in these studies[17].

### Quality of Included Studies

#### Methodological quality

Our study included 20 SRs over a 20-year span and 16 of the studies demonstrated medium quality, only four studies found high quality. No increase in SR quality, with regard to fulfillment of the AMSTAR criteria, was visible over the years, a similar finding was convinced by Monique2011[18] and Seo2012[41], but Dereck [15]and Amit [16] indicated that the number and methodological quality of trials have improved over time. Three studies [4 19 20] used a “vote-counting” method to establish general conclusions. “Vote-counting” is a method that the overall results of the trials were reported by taking the number of trials with statistically significant results and dividing them by the total number of trials[19]. This method does not consider the magnitude of effects and may have underestimated the overall efficacy[20]. Hedge concluded that aggregated outcome relied upon vote counting increasing the risk of type 2 (false negative) error and formal assessment for publication bias using funnel plots was not possible with the vote-counting technique[4]. Meanwhile, a common bias, as reported by Gillaizeau2013[30] and Walton1999[36], of the included studies was that when studies were randomized by participant, the same healthcare professional may have given treatment both to intervention and control groups. It is possible that the CDSS influenced the treatment of the control groups.

#### Reporting quality

The reporting quality of 20 SRs varied significantly. Some contents were particularly strong, such as the reporting of search of studies, selection of articles, and synthesis of results. However, there are also some realms need to be improved. One of the issues may be the insufficient reporting of publication bias. Sahota2011 reported that publication bias exists in this field and the presence of publication bias may be a significant confounding factor when authors are trying to aggregate results[19]. This problem results in studies with small sample sizes and non-significant effects being left out of the aggregated pool[42]. The second significant issue is the lack of quantitative analysis, such as meta-analysis, sensitivity or subgroup analyses, meta-regression. One of the possible reason may be the bulk of the qualitative literature[42]. Only 3 of the 20 SRs attempted to compute effect sizes, and all of those were in the domain of medication dose. The majority of included SRs (85%) used narrative descriptions or a “vote-counting” method to establish general conclusions. Another possible factor is the heterogeneity. In our study, the variation in the study design, clinical setting, study population, software specifications, and CDSS workflow integration led the results were not consistent among the 20 SRs, even when evaluating the same drug or the same disease[20]. The reporting flaws would affect the method quality, the integrity and accuracy of researches, so the quality of reporting still needs further improvement [28].

### Synthesis of the systematic reviews results

It is clear from our synthesis that most SRs (95%) measured process of care outcomes and the results indicated that the application of CDSS would improve the outcomes. Similar findings were reported by a number of published studies [4 19 20 30 31 34 36]. In addition, our study found that CDSS that alert or reminder participants’ adherence to medication were most likely to impact the medication errors and patient care by improving the process of care outcomes. The degree to which participants adherence to the computerized medication advice will vary depending on the specificity level of computer-generated advice. As reported by Monique 2011[18], the specificity level of computer-generated advice is known to highly influence the chance that physicians adhere to the advice, with low specificity resulting in computer-advice fatigue and in situations where physicians ignore the advice. Simultaneously, consistent with previous studies[17 29 32], the results of this overview suggests that CDSS has a positive effect in changing prescribing outcomes. Alldred2013 illustrated that CDSS improved the prescribing by discontinuing inappropriate medication; commencing beneficial medicines; and ensuring appropriate monitoring of long-term conditions and medicines[38]. Consequently, this process may lead to a reduction in medication error and the improvement of medication safety.

However, while 65% of the included studies measured a patient outcome, only a small proportion demonstrated positive findings. Regarding lack of positive findings on patient outcomes, the related study by Monique 2011 showed that it was likely attributed to the small sample sizes in the original studies that consequently were underpowered[18]. Furthermore, follow-up periods in some studies were always too short to assess long-term differences on patient outcomes associated with the computerized interventions[43]. Outcomes such as hospital length of stay, death and bleeding complications will be influenced by factors other than better medication management[17]. Our study also found that CDSS for medication dosing assistants were most likely to impact the medication errors and patient care by improving the related patient outcome. An explanation for the findings may be that computers help doctors to tailor drug doses more accurately to individual patients, bringing benefits for patients and reducing the time that they spent in hospital[36]. This was also elucidated in the study by Kuperman who found that computer-generated orders are more legible than those written by hand [44]. A knowledge-based CDSS can assure that the order is safe and compliant with guidelines [45] because it introduces automation at the time of ordering, a key process in health care[44].

### Strengths and Limitations

Our study has several strengths. We innovatively assessed the quality of SRs on the effectiveness of CDSS interventions using AMSTAR and PRISMA. Secondly, the literature search was comprehensive and detailed inclusion and exclusion criteria were developed to ensure transparency and reproducibility in the judgments. Thirdly, we manually searched the reference lists of the selected SRs to identify SRs that we could have missed in our literature search. In addition, the utilization of two independent reviewers for preselection of SRs, the assessment of SRs’ quality and the final data extraction would be a great help to avoid mistakes and subjective judgments.

Meanwhile, our study also has some limitations. Firstly, we were unable to use meta-analysis to pool effect sizes, given the substantial differences among the outcomes evaluated. Secondly, we defined improvement in 50% or more of the RCTs as strong evidence, in 40–50% of the RCTs as limited evidence, and in less than 40% of the RCTs or non-randomized studies as insufficient evidence respectively. The methods, combined with the strict inclusion criteria, may have underestimated the effects of CDSS intervention. Thirdly, we tried to interpret the overall effects of CDSS by aggregating all included studies, the results of high quality studies are given the same weight as low-quality studies. Fourthly, there may be some overlaps of our included studies which may have led to an overestimation the effects of CDSS. We therefore analyzed the number of unique studies from the total number included in all SRs and provided overall estimates of the evidence that CDSS significantly impacted process of care outcomes and patient outcomes [18]. Lastly, we classified the methodological quality at three levels based on the final scores of each studies, but scoring systems are controversial [46].

### Future Directions

First, larger samples and longer-term studies are required to ensure more reliable evidence base on the effects of CDSS on medication error. Decision makers should balance the effects of CDSS against its burden on costs and workflow. However, the increasing number and quality of CDSS trials in the past few years and the rapid assimilation of technological information into clinical settings bode well for the future of improving the effectiveness and efficiency of clinical care[15].

## Conclusions

CDSS reduces medication error by obviously improving process of care, but inconsistently improving patient outcomes. There is significant evidence that CDSS for medication alerts or reminders can positively impact process of care outcomes and CDSS related to medication dosing assistants were most likely to impact patient outcomes. The methodological and reporting quality of the included studies were varied. The changes of process care may be the most immediate effect of CDSS, nevertheless, like other health care interventions, CDSS must demonstrate benefit on patient outcomes related to drug efficacy and safety before extensively recommended into clinical care, particularly given the cost of implementing and maintaining computerization.

## Supporting Information

S1 Checklist PRISMA checklist. (DOC)

S1 File Search Strategy for Embase, Pubmed and Cochrane library. (DOCX)
