# Supplementary material for: The Effects of Clinical Decision Support Systems on Medication Safety: An Overview
Source: PLoS One. 2016 Dec 15;11(12):e0167683. doi: 10.1371/journal.pone.0167683 (PMC5157990; doi:10.1371/journal.pone.0167683)
Supplement: S1 File — (DOCX) [file pone.0167683.s002.docx]

**EMBASE**

| [**order**](app:ds:order) | **searches** |
| --- | --- |
| 1 | Medical order entry systems.mp. |
| 2 | Computerized order entry.mp. or exp computerized provider order entry/ |
| 3 | Computerized prescriber order entry.mp. or exp computerized provider order entry/ |
| 4 | Computerized provider order entry.mp. or exp computerized provider order entry/ or exp decision support system/ |
| 5 | Electronic order entry.mp. or exp computerized provider order entry/ |
| 6 | Electronic prescribing.mp. or exp electronic prescribing/ or exp electronic medical record/ |
| 7 | exp electronic medical record/ or Electronic physician order entry.mp. or exp decision support system/ |
| 8 | Computerized physician order entry.mp. or exp computerized provider order entry/ |
| 9 | Drug-therapy, computer-assisted.mp. or exp computer assisted drug therapy/ |
| 10 | Decision support systems, clinical.mp. or exp decision support system/ |
| 11 | Decision-support systems.mp. or exp decision support system/ |
| 12 | exp medical decision making/ or exp decision support system/ or Clinical decision-support systems.mp. or exp clinical decision making/ or exp computer assisted diagnosis/ |
| 13 | Reminder systems.mp. or exp reminder system/ |
| 14 | decision-making,computer assisted.mp. or exp decision support system/ |
| 15 | Diagnosis, computer-assisted.mp. or exp computer assisted diagnosis/ |
| 16 | Therapy, computer-assisted.mp. or exp computer assisted therapy/ |
| 17 | Expert systems.mp. or exp expert system/ |
| 18 | 1 or 2 or 3 or 4 or 5 or 6 or 7 or 8 or 9 or 10 or 11 or 12 or 13 or 14 or 15 or 16 or 17 |
| 19 | Medical order entry system*.ti. or Medical order entry system*.ab. or computerized order entry.ab. or computerized order entry.ti. or Computerized prescriber order entry.ab. or Computerized prescriber order entry.ti. or Computerized provider order entry.ab. or Computerized provider order entry.ti. or Electronic order entry.ab. or Electronic order entry.ti. or Electronic prescribing.ab. or Electronic prescribing.ti. or Electronic physician order entry.ab. or Electronic physician order entry.ti. or Computerized physician order entry.ab. or Computerized physician order entry.ti. or computer assisted drug therapy.ab. or computer assisted drug therapy.ti. or Decision support systems.ab. or Decision support systems.ti. or reminder system*.ab. or reminder system*.ti. or computer assisted decision making.ab. or computer assisted decision making.ti. or computer assisted diagnosis.ab. or computer assisted diagnosis.ti. or computer assisted therapy.ab. or computer assisted therapy.ti. or expert system*.ab. or expert system*.ti. |
| 20 | 18 or 24 |
| 21 | systematic review.mp. or exp "systematic review"/ |
| 22 | meta analysis/ |
| 23 | meta analysis.ti. or meta analysis.ab. or systematic review.ab. or systematic review.ti. |
| 24 | 19 or 20 or 21 |
| 25 | 22 and 25 |
| 26 | limit 26 to (human and english language) |

**PUBMED**

| [**order**](app:ds:order) | **searches** |
| --- | --- |
| 1 | (medical order entry systems[MeSH Terms]) OR medical order entry system*[Title/Abstract]) OR computerized order entry[Title/Abstract]) OR computerized prescriber order entry[Title/Abstract]) OR computerized provider order entry[Title/Abstract]) OR computerized physician order entry[Title/Abstract]) OR electronic order entry[Title/Abstract]) OR electronic prescribing[MeSH Terms]) OR electronic prescribing[Title/Abstract]) OR cpoe[Title/Abstract]) OR drug-therapy,computer assisted[MeSH Terms]) OR computer assisted drug therapy[Title/Abstract]) OR decision support systems, clinical[MeSH Terms]) OR decision support system*[Title/Abstract]) OR reminder system*[Title/Abstract]) OR decision-making,computer assisted[MeSH Terms]) OR computer assisted decision making[Title/Abstract]) OR diagnosis, computer assisted[MeSH Terms]) OR computer assisted diagnosis[Title/Abstract]) OR therapy, computer assisted[MeSH Terms]) OR computer assisted therapy[Title/Abstract]) OR expert systems[MeSH Terms]) OR expert system*[Title/Abstract]) OR *CDS*[Title/Abstract] |
| 2 | (literature review[Title/Abstract]) OR critical appraisal[Title/Abstract]) OR meta analysis[Publication Type]) OR systematic review[Text Word]) OR medline[Text Word] |

**Cochrane Library**

| [**order**](app:ds:order) | **searches** |
| --- | --- |
| 1 | Medical order entry systems:ti,ab,kw |
| 2 | Computerized order entry:ti,ab,kw |
| 3 | Computerized prescriber order entry:ti,ab,kw |
| 4 | Computerized provider order entry:ti,ab,kw |
| 5 | Electronic order entry:ti,ab,kw |
| 6 | Electronic prescribing:ti,ab,kw |
| 7 | Electronic physician order entry:ti,ab,kw |
| 8 | Computerized physician order entry:ti,ab,kw |
| 9 | Drug-therapy, computer-assisted:ti,ab,kw |
| 10 | Decision support systems, clinical:ti,ab,kw |
| 11 | Decision-support system:ti,ab,kw |
| 12 | Clinical decision-support systems:ti,ab,kw |
| 13 | Reminder systems:ti,ab,kw |
| 14 | Computer assisted decision-making: ti,ab,kw |
| 15 | Diagnosis, computer-assisted:ti,ab,kw |
| 16 | Therapy, computer-assisted:ti,ab,kw |
| 17 | Expert systems:ti,ab,kw |
| 18 | 1 or 2 or 3 or 4 or 5 or 6 or 7 or 8 or 9 or 10 or 11 or 12 or 13 or 14 or 15 or 16 or 17 |
